# A novel prognostic model based on three clinic-related miRNAs for prostate cancer

**DOI:** 10.3389/fsurg.2022.872953

**Published:** 2022-07-25

**Authors:** Ping Che, Shihao Jiang, Weiyang Zhang, Huixuan Zhu, Daorong Hu, Delin Wang

**Affiliations:** ^1^ Department of Urology, The First Affiliated Hospital of Chongqing Medical University, Chongqing, China; ^2^ Department of Pediatric Surgery, Maternity and Child Health Hospital of Chongqing Hechuan, Chongqing, China; ^3^ Department of Urology, The People's Hospital of Hechuan, Chongqing, China

**Keywords:** PCA, miRNA, risk model, prognosis, bioinformactics

## Abstract

**Background:**

Prostate cancer (PCa) is the second most common malignant tumor in men worldwide. MiRNAs have been reported to play significant roles in prognosis prediction for patients with malignant tumors.

**Methods:**

The survival-related miRNAs (sDMIRs) were identified by Cox regression analysis. A risk score model (RSM) was established based on three sDMIRs. The expression levels of sDMIRs in cell lines and clinical samples were detected *via* quantitative polymerase chain reaction. The correlations between sDMIRs and clinicopathological characteristics of PCa patients were evaluated using the chi-square test and Fisher's exact probability method.

**Results:**

Four sDMIRs were remarkably related to the prognosis of PCa patients based on univariate Cox analysis, of which miR-10a-5p, miR-20a-5p, and miR-508-3p were used to establish the RSM. The OS in the low-risk group was better than that in the high-risk group. In the verification of various prostate cell lines and clinical samples from 162 PCa patients, the prominently higher expression of miR-10a-5p and miR-20a-5p and lower expression of miR-508-3p were detected in PCa cell lines and tumor tissues, especially the more advanced T-stage. Besides, the higher expression of miR-20a-5p and miR-10a-5p was significantly correlated to the higher level of PSA, Gleason score, more advanced T-stage, and distant metastasis status.

**Conclusion:**

We identify and validate the clinical significance of three sDMIRs and establish a verified RSM to evaluate the prognosis for PCa patients. The findings not only provide a reliable tool for clinical decision-makers to evaluate patients' prognosis but also offer a novel perspective into the field of biomarker identification.

## Introduction

Prostate cancer (PCa) is the second most common malignant tumor in men worldwide. With aging of the population in worldwide, the incidence and mortality of PCa have increased significantly, becoming the most common cancer in the male genitourinary system in China (1, 2). Currently, a growing body of studies is exploring more promising tools for diagnosis, treatment, and prognosis prediction of PCa (3).

With further insight into the significance of microRNAs (miRNAs), a class of 19- to 24-nucleotide noncoding RNAs, more studies unravel their prominent roles in cellular processes and various pathologies (4, 5). Importantly, emerged as an attractive approach for clinical application, miRNAs not only are considered as biomarkers for diagnosis and prognosis or in the prediction of therapy response but also as therapeutic tools (6, 7). Therefore, identifying promising miRNAs and recognizing their potential in diseases may provide more options for clinical decision-makers. It has been suggested that miRNAs regulate tumorigenesis and development and affect the prognosis of different tumors (4). Weiss et al. illuminated that miR-182-5p and miR-205-5p as potential biomarkers for p16-positive oropharyngeal squamous cell carcinoma can help to further stratify patients (8). miR-7 was demonstrated to inhibit epithelial–mesenchymal transition and invasiveness in glioblastoma multiforme by targeting T-Box 2 (9). Wu et al. recapitulated that miR-424 and miR-503, as a cluster concomitantly expressed in several cancer cells, are dysregulated and play intricate roles in tumor initiation and progression, involving various targets and molecular mechanisms (10). Long noncoding RNA LEF1-AS1-regulated miR-10a-5p was reported to promote chemoresistance in hepatocellular carcinoma cells by activating the AKT signaling pathway (11). Worst et al. found that miR-10a-5p and miR-29b-3p were highly expressed in the blood of PCa patients and could serve as potential new PCa detection markers (12). Cheng et al. revealed that miR-20a-5p promotes colorectal cancer metastasis through downregulating Smad4 (13). Few reports have studied the roles of miR-508-3p, especially in cancer. Guo et al. demonstrated that miR-508-3p suppresses the progression of ovarian carcinoma by regulating expression of CCNA2 and MMP7 (14). However, whether miR-508-3p, miR-20a-5p, and miR-10a-5p affect PCa occurrence and development or are considered biomarkers for patients with PCa needs to be further demonstrated.

In the present study, we attempt to establish a risk score model (RSM) with clinical significance based on the differential expression of miRNAs in PCa and investigate its prominent roles in predicting the prognosis of patients with PCa. The findings will open up novel avenues for exploring the underlying mechanisms of miRNAs in PCa, and the risk score model will offer another perspective for clinical strategy making.

## Methods

### Clinical prostate samples

A total of 162 clinical samples of PCa tissues and adjacent normal tissues were acquired from the patients diagnosed with PCa and accepted operation in the First Affiliated Hospital of Chongqing Medical University from January 2019 to September 2021. The acquired samples of PCa patients were frozen in liquid nitrogen immediately and then stored at −80 °C until miRNA extraction.

### Data downloading and analysis of differential mRNAs and differential miRNAs

Transcriptome RNA sequencing and miRNA data of PCa were downloaded from the TCGA data portal (https://portal.gdc.cancer.gov/), which contained data from 51 nontumor samples and 489 PCa samples. Clinical data of these PCa patients were downloaded and extracted. MiRNA and mRNA data were combined into matrix files by merging scripts of the Perl language (http://www.perl.org/). The limma package of R software (https://bioconductor.org/packages/release/bioc/html/limma.html) was used to screen for differentially expressed miRNAs and mRNAs in PCa and adjacent normal tissues. We performed differential analysis on all mRNA and miRNA data with the screening value of “FDR < 0.05, log| FC |  > 1, and *p* < 0.05”.

### Survival-related DMIRs and creation of the RSM

DEMIRs related to clinical prognosis values in PCa patients were selected as survival-related DMIRs (sDMIRs). Univariate Cox analysis was used to screen sDMIRs (*p* < 0.05). We determined hazard ratio (HR) to separate sDMIRs into protective and deleterious parts. sDMIRs were further analyzed by the multivariate Cox analysis (*p* < 0.05). To detect the clinical value of sDMIRs, we divided PCa patients into a high-risk group and a low-risk group based on the median risk score. We established RSM using the expression data multiplied by Cox regression coefficients. The formula is as follows: [Expression level of hsa-miR-10a-5p * (0.877309)] + [Expression level of hsa-miR-20a-5p * (0.792393)] + [Expression level of hsa-miR-508-3p * (−0.596601)].

## The genes of sDMIRs and bioinformatics analysis

The target genes were selected by the TargetScan (http://www.targetscan.org/vert_72/), miRTarBase (http://mirtarbase.mbc.nctu.edu.tw/php/index.php), and miRDB (http://mirdb.org/) databases. The filter standard of a target gene is no less than two databases supported. Cytoscape software version 3.7.2 was used to show the results of the miRNA–mRNA regulation network. Functional enrichment analysis was performed through the Gene Ontology (GO) and Kyoto Encyclopedia of Genes and Genomes (KEGG) pathways to explore the underlying molecular mechanisms of sDMIRs. GO and KEGG pathways were analyzed by cluster profiler, org.Hs.eg.db, and enrichplot of R software. The receiver operating characteristic (ROC) curves were drawn by the survival ROC package of R software. Univariate Cox regression analysis and multivariate Cox regression analysis were used to select the sDMIRs. We drew Kaplan–Meier curves to illustrate the OS of the high-risk group and the low-risk group of PCa patients.

### Real-time quantitative PCR

Total RNAs were extracted from the PCa tumor and adjacent normal tissues by TRIzol (Takara, Beijing, China). RNAs were reverse-transcribed to cDNA by the PrimeScript RT reagent kit (Takara, Beijing, China). The reaction steps are as follows: 37°C for 15 min and 85°C for 5 s. The quantitative polymerase chain reaction (qPCR) was carried out on an ABI 7500 Real-Time PCR System (Applied Biosystems, Shanghai, China) by SYBR Green assay (Takara, Beijing, China). The reaction cycling conditions were 95°C for 30 s, 45 cycles of 95°C for 5 s, and 60°C for 30 s. The primer sequences are shown in Table 1. The relative quantification values of miRNAs were standardized to U6 using the −ΔΔ 2 Ct method.

**Table 1:** Primer sequences of hsa-miR-10a-5p, hsa-miR-20a-5p, and hsa-miR-508-3p.

hsa-miR-10a-5p	F primer (5′–3′)	CGCGTACCCTGTAGATCCGAA
	R primer (5′–3′)	GTCGTATCCAGTGCAGGGTC
	RT (5′–3′)	GTCGTATCCAGTGCAGGGTCCGAGGTATTCGCACTGGATACGACCACAAA
hsa-miR-20a-5p	F primer (5′–3′)	CTGCGCGTAAAGTGCTTATAGTG
	R primer (5′–3′)	GTCGTATCCAGTGCAGGGTC
	RT (5′–3′)	GTCGTATCCAGTGCAGGGTCCGAGGTATTCGCACTGGATACGACCTACCT
hsa-miR-508-3p	F primer (5′–3′)	GCGTGATTGTAGCCTTTTGGAGT
	R primer (5′-3′)	GTCGTATCCAGTGCAGGGTC
	RT (5′–3′)	GTCGTATCCAGTGCAGGGTCCGAGGTATTCGCACTGGATACGACTCTACT
U6	F primer (5′–3′)	CTCGCTTCGGCAGCACA
	R primer (5′–3′)	AACGCTTCACGAATTTGCGT
	RT (5′–3′)	AAAATATGGAACGCTTCACGAATTTG

F primer: forward primer; R primer: reverse primer; RT: reverse transcription.

### Ethics statement

Informed consent forms were signed by all patients before the study. The research protocol has been approved by the Ethics Committee of the First Affiliated Hospital of Chongqing Medical University and is based on the ethical principles of medical research involving human subjects in the Helsinki Declaration.

### Statistical analysis

All statistical analyses were conducted by SPSS22.0 software (SPSS Inc., Chicago, IL) and GraphPad Prism8 (GraphPad Software Inc., La Jolla, CA). The clinical correlations were determined by ANOVA, a *post hoc* test (Bonferroni's method), and an independent *t*-test. The correlations between miRNA expression levels and clinicopathological characteristics of patients with PCa were evaluated using the chi-square test and Fisher's exact probability method. *p* < 0.05 was considered statistically significant.

## Results

### Differentially expressed miRNAs and mRNAs

**Figure 1:**
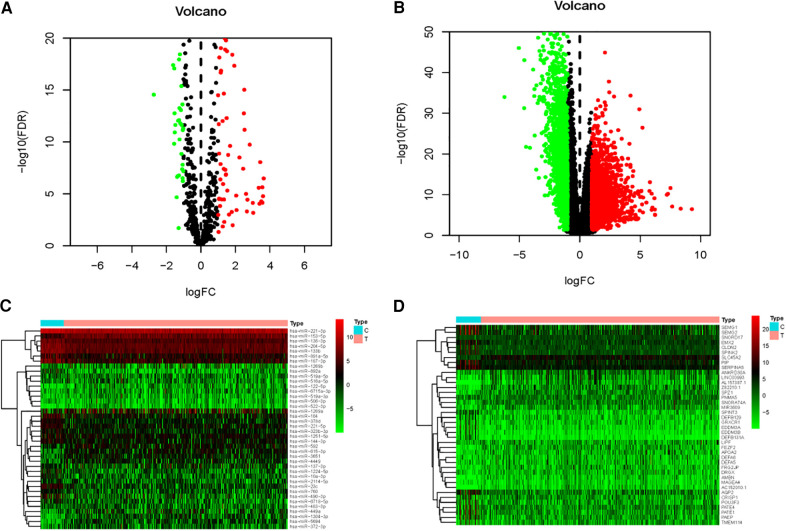
Differentially expressed miRNAs and mRNAs of PCa. Volcano plot (**A**) and heatmap (**C**) showing the differential expression miRNAs of PCa tissues and adjacent-normal tissues. Volcano plot (**B**) and heatmap (**D**) demonstrating differentially expressed mRNAs of PCa and adjacent tissues. FDR < 0.05, log_2_ | FC | > 1 and *p* < 0.05.

A total of 132 differentially expressed PCa miRNAs were selected by the limma package of R software, of which 56 were downregulated and 76 were upregulated (Figure 1A). The 50 most upregulated and downregulated miRNAs were respectively demonstrated by the heatmap (Figure 1C). We further confirmed 4161 differentially expressed mRNAs, including 2032 downregulated and 2129 upregulated mRNAs (Figure 1B). The most 50 upregulated and downregulated mRNAs were shown in the heatmap (Figure 1D).

### Identification of survival-related DMIRs and establishment of the risk score model

**Figure 2:**
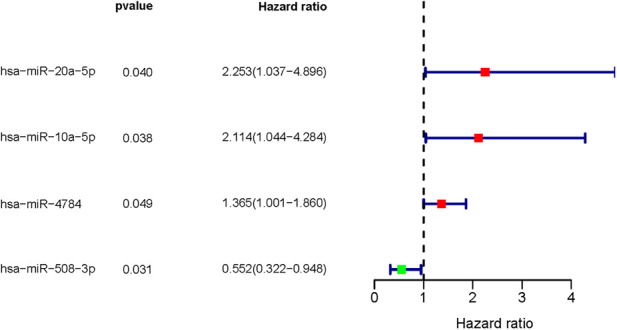
Survival-related differentially expressed miRNAs. Forest plot showing the survival-related values (hazard ratios) of DMIRs (hsa-miR-20a-5p, hsa-miR-10a-5p, hsa-miR-4784, and hsa-miR-508-3p). The red portion represents upregulated sDMIRs, and the green portion represents downregulated sDMIRs.

**Figure 3:**
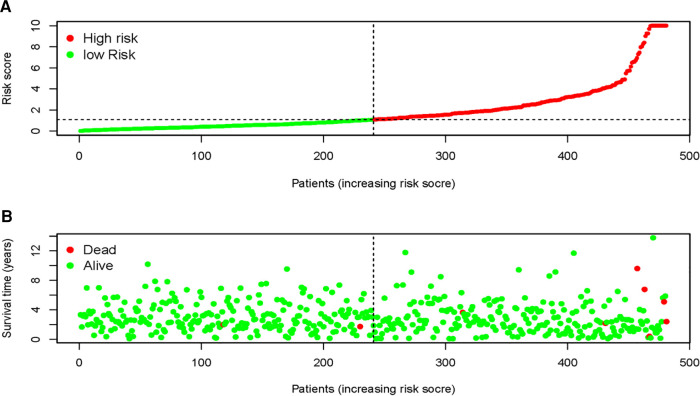
Risk score model (RSM) constructed by sDMIRs. risk score distribution in the high-risk group and the low-risk group of PCa patients (**A**). Survival status between the high-risk group and the low-risk group of PCa patients (**B**).

**Figure 4:**
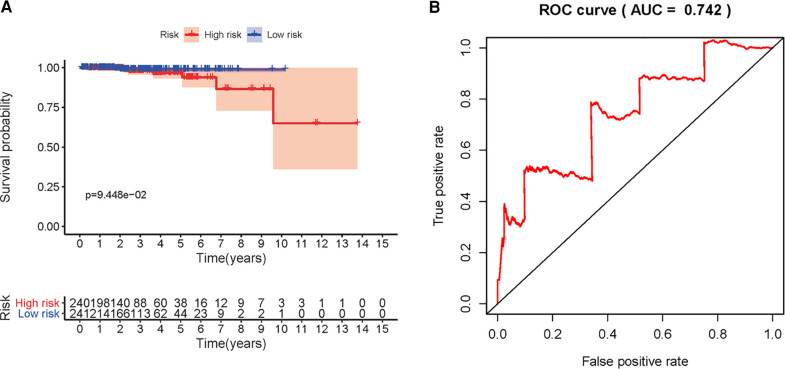
Survival curve and receiver operating characteristic (ROC) curve of RSM. **(A)** Kaplan–Meier survival curve of OS in low-risk and high-risk groups of PCa patients. The high-risk group demonstrated a poor prognosis for PCa patients. **(B)** ROC curves indicated the accuracy and sensitivity of RSM. The AUC was 0.742.

We next explored whether the DMIRs were correlated with the prognosis of PCa patients. We detected four sDMIRs by univariate Cox regression analysis. As shown in Figure 2, miR-508-3p demonstrated a positive correlation with poor prognosis, while miR-20a-5p, miR-10a-5p, and miR-4784 illustrated reversed results. To establish the risk score model, three sDMIRs (miR-10a-5p, miR-20a-5p, and miR-508-3p) were screened by the multivariate Cox regression analysis (*p* < 0.05). Moreover, the survival curves of these sDMIRs demonstrated that the higher expression levels of sDMIRs were correlated with poor prognosis except for miR-508a-3p. (Supplementary Figure 1A–C). The selected three sDMIRs were selected to establish the RSM, of which the PCa patients were divided into a high-risk group and a low-risk group based on the median risk score (Figure 3A). The mortality rate enhanced significantly in the high-risk group (Figure 3B). The survival probabilities of patients in the low-risk group were remarkably higher than that of the high-risk group (Figure 4A). The ROC curve was used to investigate the sensitivity of the risk score model, and the AUC of the ROC curve was 0.742, highlighting the satisfied sensitivity of RSM based on the three sDMIRs in predicting the prognosis (Figure 4B).

###  The relationships between the RSM and clinical features

**Figure 5:**
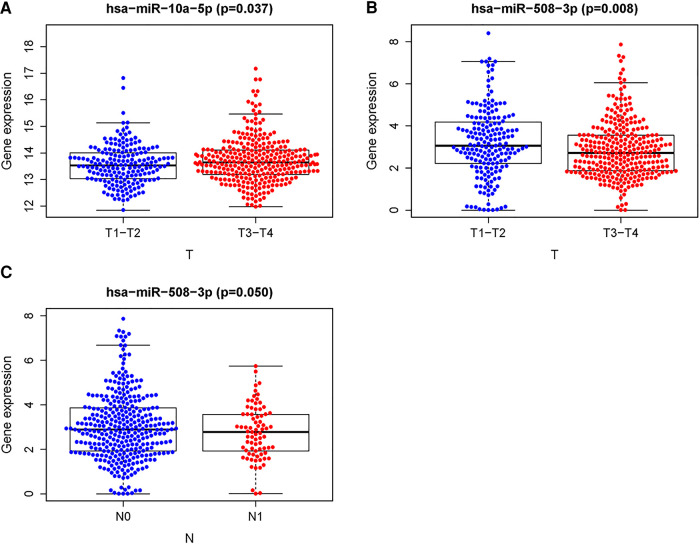
Relationships between the sDMIRs and the clinical features. Relationships between the hsa-mir-10a-5p (**A**), hsa-mir-508-3p (**B)**, and T-stages, N-stages (**C**).

The relationships between the RSM and clinical features of PCa were detected by an independent *t*-test. We found that patients with advanced T-stages (Figure 5A) were correlated with remarkably higher expression of miR-10a-5p. High expression of miR-508-3p was observed in early T-stages (Figure 5B). The univariate Cox regression and multivariate Cox regression analyses were displayed to verify further whether the risk score could serve as an independent prognostic factor. As shown in Table 2, the risk score was related to prognosis by the univariate Cox regression analysis. The results of the multivariate Cox regression analysis also showed that the risk score has the potential for predicting the prognosis of PCa patients. These results highlighted that the risk score could be a sensitivity and accuracy independent prognostic factor for Pca patients.

**Table 2:** Univariate and multivariate analyses of PCa.

Variables	Univariate analysis				Multivariate analysis			
	HR	HR 95% low	HR 95% high	*p* value	HR	HR 95% low	HR 95% high	*p* value
Age	1.063044	0.955996	1.182079	0.258912	1.081508	0.974504	1.200261	0.140454
T-stage	2.036654	0.466872	8.884564	0.343915	1.087560	0.168208	7.031675	0.929764
N-stage	3.422780	0.759087	15.43356	0.109319	5.886936	0.946026	36.63324	0.05737
Risk score	1.110030	1.053835	1.169223	8.209e-05	1.123380	1.059782	1.190793	9.125e-05

HR: hazard ratio.

### Target genes of sDMIRs and their functional enrichment analysis

**Figure 6:**
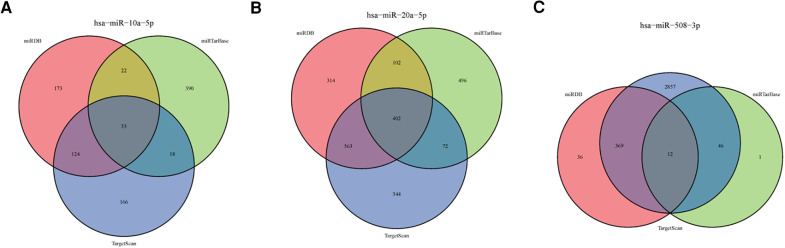
Target genes of the sDMIRs. Venn diagrams showing the target genes of the sDMIRs predicted from TargetScan, miRDB, and miRTarBase: (**A)** hsa-miR-10a-5p, (**B**) hsa-miR-20a-5p, and (**C**) hsa-miR-508-3p.

**Figure 7:**
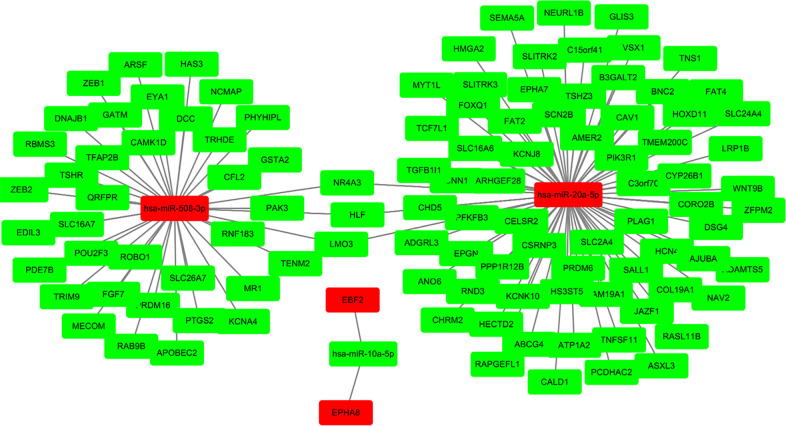
Relationships between the sDMIRs and target genes. The sDMIRs and target gene regulatory network. Red parts represent upregulated genes, and green parts represent downregulated genes.

**Figure 8:**
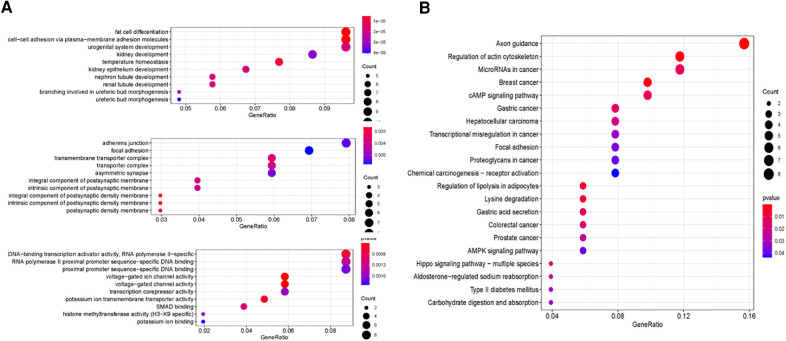
Functional enrichment analysis of target genes. The top pathways of target genes were demonstrated in biological process, cellular component, molecular function (**A**), and KEGG pathway (**B**).

To further find the regulatory relationships between sDMIRs and their target genes, we selected the target genes of sDMIRs from TargetScan, miRTarBase, and miRDB databases, and the results were demonstrated in the Venn diagrams (Figures 6A–C). Meanwhile, the regulatory networks of target genes and sDMIRs are shown in Figure 7. Some of the target genes had a significant correlation with the prognosis of Pca patients, and we further detected the survival curve of these target genes. The results showed that the higher expression levels of JAZF1, PRDM6, RBMS3, and TSHR were correlated with the poor prognosis of Pca patients (Supplementary Figure 2). We used Gene Ontology (GO) and Kyoto Encyclopedia of Genes and Genomes (KEGG) pathway analyses to investigate the potential molecular mechanisms of these target genes. The functional enrichment analysis results showed that “fat cell differentiation,” “adherens junction,” and “DNA-binding transcription activator activity, RNA polymerase II-specific” were the most enriched in biological processes (BP), cellular components (CC), and molecular functions (MF), respectively (Figure 8A). “Axon guidance” was the most enriched pathway in the KEGG pathways (Figure 8B).

### The expression of miR-508-3p, miR-20a-5p, and miR-10a-5p in prostate cell lines and tissues

**Figure 9:**
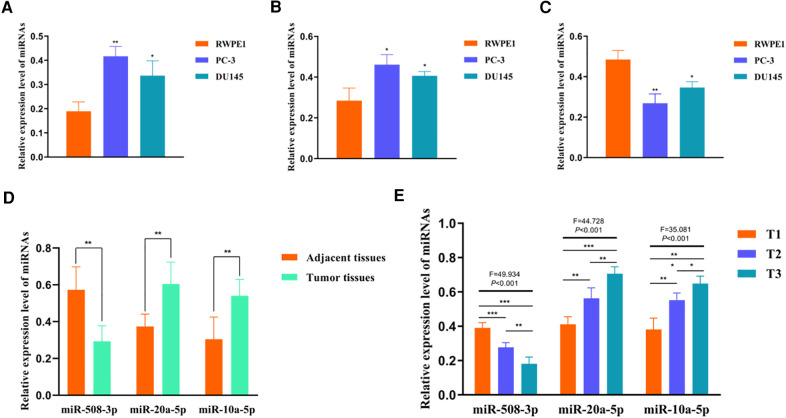
Expression levels of sDMIRs and their correlations with T-stage. The expression levels of miR-10a-5p (**A**) and miR-20a-5p (**B**) significantly decreased in prostate epithelial cell line RWPE1, but miR-508-3p (**C**) exhibited the opposite results. miR-10a-5p and miR-20a-5p were highly expressed in tumor tissues and the expression of miR-508-3p was enhanced in adjacent normal tissues (**D**). Expression level of miR-508-3p rather than miR-10a-5p decreased in the advanced T-stage (**E**).

To investigate the expression levels of sDMIRs in different prostate cell lines and clinical samples, we detected the expression of miR-508-3p, miR-20a-5p, and miR-10a-5p in nontumorigenic human prostate epithelial cell line (RWPE1), PCa cell lines (PC-3 and DU145), and the tumor tissues and adjacent normal tissues of PCa. Compared to RWPE1, the expression levels of miR-10a-5p (Figure 9A) and miR-20a-5p (Figure 9B) were obviously higher in PCa cell lines (PC-3 and DU145) but miR-508-3p (Figure 9C) showed the opposite result. Besides, we found that the expression of miR-508-3p was prominently lower in tumor tissues than that in the paired adjacent normal tissues, but we detected higher expression levels of miR-20a-5p and miR-10a-5p in tumor tissues (Figure 9D).

### The clinical correlations of miR-508-3p, miR-20a-5p, and miR-10a-5p

To further determine the clinical significance of these sDMIRs, we next examined the associations of miR-508-3p, miR-20a-5p, and miR-10a-5p with T-stage. As illustrated in Figure 9E, miR-20a-5p and miR-10a-5p expressed increasingly as the more advanced T-stages, but the expression of miR-508-3p altered inversely. Furthermore, we investigated the clinical significance of these sDMIRs by analyzing their associations with different clinicopathological characteristics. As shown in Table 3, the higher expression of miR-20a-5p and miR-10a-5p remarkably correlated to the high level of prostate-specific antigen (PSA) and Gleason score, as well as the more advanced T-stage and distant metastasis status.

**Table 3:** Relationship between miR-20a-5p and miR-10a-5p expression levels and clinicopathologic factors of BCa patients.

Parameter	*N*	Average expression of sDMIRs		*p* value
		Low	High	
Age (years)				0.341
<65	92	49	43	
≥65	70	32	38	
PSA (ng/ml)				<**0**.**001**
≤10	57	44	13	
>10	105	37	68	
Gleason score				**0**.**015**
≤6	46	30	16	
>6	116	51	65	
T-stage				<**0**.**001**
T1	25	21	4	
T2	74	34	30	
T3	63	16	47	
Lymph node status				0.386
Negative	149	73	76	
Positive	13	8	5	
Distant metastasis status				**0**.**026**
Negative	113	63	50	
Positive	49	18	31	

The bold number represents the p-values with significant differences; PSA: prostate-specific antigen.

## Discussion

Owing to new techniques in transcriptomics and genomics, our understanding of new roles and new mechanisms for miRNAs grows progressively. Better knowledge in this area will help to identify and explore more potential of miRNAs with clinical significance. Therefore, there is a growing appreciation of the roles of miRNAs in the diagnosis, treatment, and prognosis prediction of cancers, as evidenced by the increase in the number of studies on this topic recently. Zheng et al. reported that miR-4651 inhibits nonsmall-cell lung cancer cell progression by targeting bromodomain-containing protein 4 (15). MiR-1246 was demonstrated to promote the progression of hepatocellular carcinoma through activating the RORα-Wnt/β-Catenin axis (16). Barbier et al. revealed that miR-579-3p participates in the abiraterone-induced SLCO1B3 expression in PCa and further elucidated the mechanisms of abiraterone resistance (17). Byun et al. identified that the urinary microRNA-1913 to microRNA-3659 expression ratio is potentially a noninvasive diagnostic biomarker for PCa (18). Circulating miR-940 was illuminated to not only play a prominent role in PCa progression but is also a novel biomarker for PCa detection (19).

In the present study, we accessed mRNAs and miRNAs data of 540 PCa patients in TCGA. Subsequently, we screened four sDMIRs that were correlated with the prognosis of PCa patients significantly, of which three sDMIRs were further selected to construct the RSM. In the RSM, the survival probability of the high-risk group was significantly shorter than that in the low-risk group. The ROC curve of RSM was 0.742, which presented that the accuracy of our RSM was high. Compared with the individual miRNA, as Worst et al. reported (12), the present model will more accurately predict the prognosis of PCa patients and better evaluate the clinical stage. In the regulatory networks of target genes of the sDMIRs, we found that JAZF1, PRDM6, RBMS3, and TSHR were correlated with the poor prognosis of PCa patients. Moreover, in the gene set enrichment analysis, “fat cell differentiation,” “adherens junction,” and “DNA-binding transcription activator activity, RNA polymerase II-specific” were the most enriched mechanisms in BP, CC, and MF, respectively. “Axon guidance” was the top pathway in the KEGG pathways analysis. The potential mechanisms in the enrichment analysis were also demonstrated to play crucial roles in PCa progression. For example, Ramanand found that the RNA polymerase II-associated chromatin interactions, as the critical determinant, regulate aberrant transcription in PCa (20). Li et al. demonstrated that miR-96-5p hampered the expression of adherens junction-associated protein-1 and inhibited PCa cell proliferation and metastasis (21). In the study, we found that the expression of miR-10a-5p and miR-20a-5p rather than miR-508-3p was prominently enhanced in PCa cells. To further detect the expression and clinical relevance of sDMIRs, we recruited a great number of PCa patients. Finally, we validated that miR-10a-5p and miR-20a-5p were highly expressed in PCa tumor tissues and advanced T-stage, while miR-508-3p showed the opposite results.

Although we have validated the clinical significance of RSM for prognosis evaluation of PCa patients and detected the expression and clinical correlation of the sDMIRs that were used to establish the RSM, there are still some limitations that need to be overcome in future research studies. First, multiple omics analysis and more basic experiments should be performed to further uncover the underlying mechanisms of these sDMIRs. Then, *in vivo* and *in vitro* experiments of other sDIMRs should also be completed.

## Conclusion

In this study, we find and verify the clinical significance of the RSM based on three survival-related miRNAs, especially in predicting prognoses of patients with PCa. The results will provide a new and accurate model for clinical decisions and offer a novel perspective into the field of biomarkers identification.

## Data Availability

The raw data supporting the conclusions of this article will be made available by the authors, without undue reservation.

## References

[1] Cackowski F Heath E. Prostate cancer dormancy and recurrence. Cancer Lett. (2021) 1:103–8. 10.1016/j.canlet.2021.09.037 PMC8694498 34624433

[2] Yamada Y Beltran H. The treatment landscape of metastatic prostate cancer. Cancer Lett. (2021) 519:20–9. 10.1016/j.canlet.2021.06.010 34153403 PMC8403655

[3] Hou Z Huang S Li Z. Androgens in prostate cancer: a tale that never ends. Cancer Lett. (2021) 516:1–12. 10.1016/j.canlet.2021.04.010 34052327

[4] Wang L Liu L Jiang B. Dysregulation of microRNAs in metal-induced angiogenesis and carcinogenesis. Semin Cancer Biol. (2021) 76:279–86. 10.1016/j.semcancer.2021.08.009 PMC8627485 34428550

[5] Roffel M Boudewijn I van Nijnatten J Faiz A Vermeulen C van Oosterhout A Identification of asthma associated microRNAs in bronchial biopsies. Eur Respir J. (2021) 59:20194. 10.1183/13993003.01294-2021 34446467

[6] Labbé M Hoey C Ray J Potiron V Supiot S Liu S microRNAs identified in prostate cancer: correlative studies on response to ionizing radiation. Mol Cancer. (2020) 19:63. 10.1186/s12943-020-01186-6 32293453 PMC7087366

[7] Kanagasabai T Li G Shen T Gladoun N Castillo-Martin M Celada S MicroRNA-21 deficiency suppresses prostate cancer progression through downregulation of the IRS1-SREBP-1 signaling pathway. Cancer Lett. (2021) 525:46–54. 10.1016/j.canlet.2021.09.041 PMC12419200 34610416

[8] Weiss B Anczykowski M Ihler F Bertlich M Spiegel J Haubner F MicroRNA-182-5p and microRNA-205-5p as potential biomarkers for prognostic stratification of p16-positive oropharyngeal squamous cell carcinoma. Cancer Biomark. (2021). 10.3233/cbm-203149 PMC12364152 34542062

[9] Pan C Chan K Chen C Jan C Liu M Lin C MicroRNA-7 targets T-Box 2 to inhibit epithelial–mesenchymal transition and invasiveness in glioblastoma multiforme. Cancer Lett. (2020) 493:133–42. 10.1016/j.canlet.2020.08.024 32861705

[10] Wu Y Wang W Yang A Zhang R. The microRNA-424/503 cluster: a master regulator of tumorigenesis and tumor progression with paradoxical roles in cancer. Cancer Lett. (2020) 494:58–72. 10.1016/j.canlet.2020.08.027 32846190

[11] Gao J Dai C Yu X Yin X Zhou F. Long noncoding RNA LEF1-AS1 acts as a microRNA-10a-5p regulator to enhance MSI1 expression and promote chemoresistance in hepatocellular carcinoma cells through activating AKT signaling pathway. J Cell Biochem. (2021) 122:86–99. 10.1002/jcb.29833 32786108

[12] Worst TS Previti C Nitschke K Diessl N Gross JC Hoffmann L miR-10a-5p and miR-29b-3p as extracellular vesicle-associated prostate cancer detection markers. Cancers (Basel). (2019) 12(1):43. 10.3390/cancers12010043 PMC7017198 31877768

[13] Cheng D Zhao S Tang H Zhang D Sun H Yu F MicroRNA-20a-5p promotes colorectal cancer invasion and metastasis by downregulating Smad4. Oncotarget. (2016) 7:45199–213. 10.18632/oncotarget.9900 27286257 PMC5216716

[14] Guo F Zhang K Li M Cui L Liu G Yan Y Mir-508-3p suppresses the development of ovarian carcinoma by targeting CCNA2 and MMP7. Int J Oncol. (2020) 57:264–76. 10.3892/ijo.2020.5055 32377701 PMC7252466

[15] Zheng J Zhang Y Cai S Dong L Hu X Chen M MicroRNA-4651 targets bromodomain-containing protein 4 to inhibit non-small cell lung cancer cell progression. Cancer Lett. (2020) 476:129–39. 10.1016/j.canlet.2020.02.018 32081805

[16] Huang J Fu Y Gan W Liu G Zhou P Zhou C Hepatic stellate cells promote the progression of hepatocellular carcinoma through microRNA-1246-RORα-Wnt/β-catenin axis. Cancer Lett. (2020) 476:140–51. 10.1016/j.canlet.2020.02.012 32061951

[17] Barbier R McCrea E Lee K Strope J Risdon E Price D Abiraterone induces SLCO1B3 expression in prostate cancer via microRNA-579-3p. Scientific reports. (2021) 11:10765. 10.1038/s41598-021-90143-4 34031488 PMC8144422

[18] Byun Y Piao X Jeong P Kang H Seo S Moon S Urinary microRNA-1913 to microRNA-3659 expression ratio as a non-invasive diagnostic biomarker for prostate cancer. Investig Clin Urol. (2021) 62:340–8. 10.4111/icu.20200488 PMC8100013 33834642

[19] Rajendiran S Maji S Haddad A Lotan Y Nandy R Vishwanatha J MicroRNA-940 as a potential serum biomarker for prostate cancer. Front. Oncol. (2021) 11:628094. 10.3389/fonc.2021.628094 33816263 PMC8017318

[20] Ramanand SG Chen Y Yuan J Daescu K Lambros MB Houlahan KE The landscape of RNA polymerase II-associated chromatin interactions in prostate cancer. J Clin Invest. (2020) 130:3987–4005. 10.1172/jci134260 32343676 PMC7410051

[21] Li R Chen Y Wu J Cui X Zheng S Yan H LncRNA FGF14-AS2 represses growth of prostate carcinoma cells via modulating miR-96-5p/AJAP1 axis. J Clin Lab Anal. (2021) 35:e24012. 10.1002/jcla.24012 34655124 PMC8605114

