# Supplementary figures and images for: A novel prognostic model based on three clinic-related miRNAs for prostate cancer
Source: Front Surg. 2022 Jul 25;9:872953. doi: 10.3389/fsurg.2022.872953 (PMC9357906; doi:10.3389/fsurg.2022.872953)

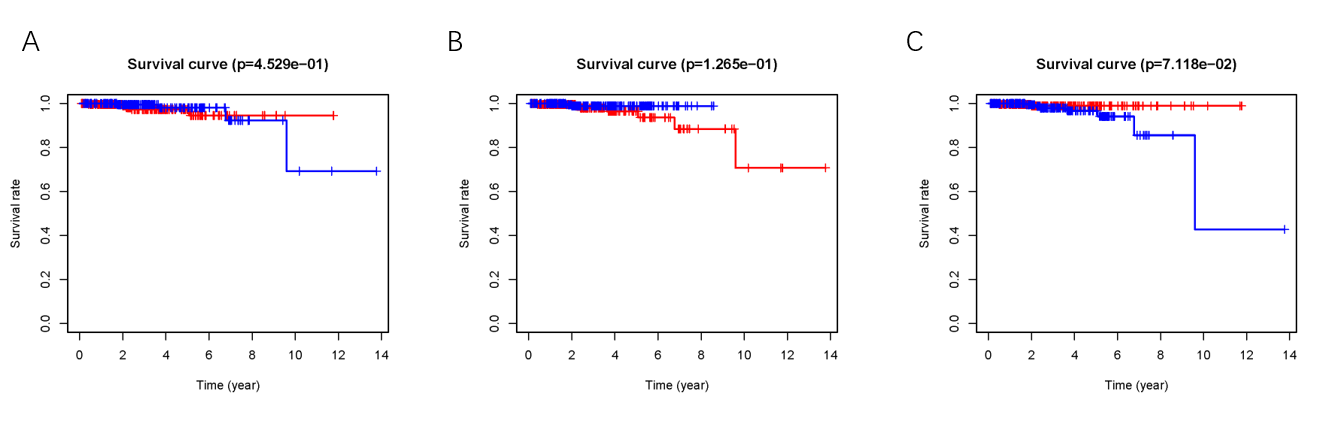

Supplement: Supplementary file 1 [file Image_1_v1.tif]

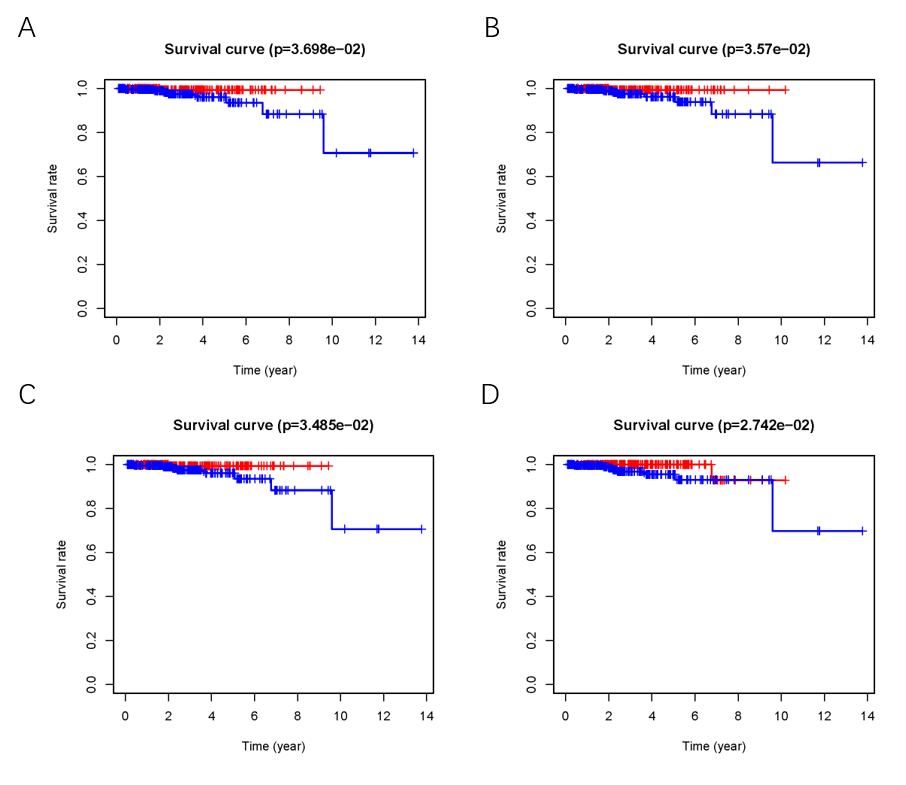

Supplement: Supplementary file 2 [file Image_2_v1.tif]
